# Determinants of suboptimal long-term secondary prevention of acute myocardial infarction: the structural interview method and physical examinations

**DOI:** 10.1186/s12872-019-1238-5

**Published:** 2019-11-06

**Authors:** Maria Sakalaki, Salim Barywani, Annika Rosengren, Lena Björck, Michael Fu

**Affiliations:** 1 Department of Molecular and Clinical Medicine, Institute of Medicine, Sahlgrenska Academy, University of Gothenburg, Gothenburg, Sweden; 2 Region Västra Götaland, Department of Medicine, Geriatrics and Emergency Medicine, Sahlgrenska University Hospital/Östra, Gothenburg, Sweden

**Keywords:** Cardiovascular disease, Secondary prevention, Myocardial infarction, Diabetes

## Abstract

**Background:**

Secondary prevention after an acute myocardial infarction (AMI) reduces morbidity and mortality, but suboptimal secondary prevention of cardiovascular disease is common. Therefore, the present study aimed to identify potential underlying factors for suboptimal secondary prevention 2 years after an AMI event.

**Methods:**

Patients aged 18–85 years at the time of their index AMI and hospitalized between July 2010 and December 2011, were identified retrospectively and consecutively from hospital discharge records. All patients who agreed to participate underwent a structured interview, physical examinations and laboratory analysis 2 years after their index AMI. The secondary preventive goals included are; blood pressure < 140/90 mmHg, LDL < 1.8 mmol/L, HbA1c < 48 mmol/mol, regular physical activity that causes sweating at least twice a week, non-smoking and BMI < 25 kg/m^2^. Multivariable and univariable logistic regression models were applied to identify independent predictors of different secondary prevention achievements.

**Results:**

Of the 200 patients (mean age 63.3 ± 9.7 years) included in the study, 159 (80%) were men. No common determinants were found in patients who failed to achieve at least six secondary prevention guideline-directed goals. For individual secondary prevention goals, several determinants were defined. Patients born in Sweden were less likely to achieve optimal lipid control [odds ratio (OR) 0.28 (95% confidence interval, CI 0.12–0.63)]. Younger (≤ 65 years) [OR 0.24 (95% CI 0.07–0.74)] and unemployed patients [OR 0.23 (95% CI 0.06–0.82)] were less likely to be non-smokers. Patients with diabetes mellitus [OR 0.21 (95% CI 0.04–0.98)] or with a walking aid [OR 0.23 (95% CI 0.07–0.71)] were less likely to achieve an optimal body mass index (BMI < 25). Living alone was an independent predictor of achieving regular physical activity [OR 1.94 (95% CI 1.02–3.69)].

**Conclusion:**

Long-term secondary prevention remained suboptimal 2 years after an AMI. Causes are likely multifactorial, with no single determinant for all six guideline-recommended preventive goals. Therefore a tailored comprehensive assessment should be requested and updated and treatment of risk factors should be applied.

## Background

Cardiovascular disease (CVD) is the leading cause of death and disease burden globally. Persons at risk for CVD or who are already affected are in urgent need of preventive counselling and medication [1]. Guidelines for CVD prevention call upon lifestyle changes, such as heightened attention to diet and exercise, attainment of normal body mass index (BMI), smoking cessation and controlling risk factors (blood pressure, glucose levels, cholesterol), complemented with evidence-based cardiovascular medication. The overall cardiovascular risk remains high after a myocardial infarction, which warrants long-term active prevention and follow-up [2, 3]. Therefore, it is of importance to fulfill these evidence-based recommendations for secondary prevention after an acute CVD event to lower morbidity and mortality and improve quality of life [4, 5].

Several studies have shown that secondary prevention after acute myocardial infarction (AMI) is suboptimal [6–9]. In a Swedish study [10] our team has shown that only 3.5% of patients with AMI fulfilled all six predefined goals for optimal secondary prevention 2 years after an AMI. Only 18.5% reached a low-density-lipoprotein (LDL) level < 1.8 mmol/L and 57.0% a systolic blood pressure < 140 mmHg^10^. In another Swedish study 46% of the participants with established coronary heart disease (CHD) had elevated blood pressure, 29% had elevated LDL and 79% were overweight or obese [11]. Still, a recently published comparison between the EUROASPIRE (European Action on Secondary and Primary Prevention by Intervention to Reduce Events) surveys II, III and IV showed improvement in blood pressure and lipid control, although with increasing rates of obesity and diabetes and persisting high rates of smoking [12].

To accomplish an optimal secondary prevention after AMI it is necessary to define determinants that can influence preventive measures. Therefore, our study aimed to identify the underlying factors for suboptimal secondary prevention 2 years after an AMI.

## Methods

### Study population

This study is a subgroup analysis from the prospective SEPAT study, which has been previously described [10]. Briefly, patients between the ages of 18–85 years and hospitalized with AMI between July 2010 and December 2011 and still alive 2 years after the index event were included [10].

### Structured interview

The individual interview was led by our research nurses at a minimum of 2 years after the index AMI event [10]. All information on education, living conditions, marital status, medical history, medication, symptoms, mental health, tobacco use, alcohol use and physical activity were collected through structured interview with each participant by our research nurses.

### Definitions

Employment was coded as working or not working and higher education as having a university degree. Smoking cessation after the AMI included patients that had quit smoking. BMI was calculated as weight (kg)/height (m) squared. Overweight was defined as BMI ≥25 kg/m^2^. Walking difficulty was defined as having the need to use any kind of mobility aid (cane, walker, walker cane hybrid, gait trainers, wheelchair). Hyperlipidemia was defined as total cholesterol > 6.2 mmol/L or using lipid-lowering medication. Diabetes was defined as fasting plasma glucose > 7 mmol/L or using oral medication, insulin or both.

The definition of depression was feeling depressed for more than 2 consecutive weeks during the past 12 months. Self-perceived stress was categorized into three levels: 1) several episodes of stress during the past 5 years, 2) persistent stress during the past year and 3) persistent stress during the past 5 years [13]. Physical activity during leisure time was defined as following; sedentary, moderate physical activity at least 2 h per week (without sweating), regular physical activity (1–2 times per week) and vigorous physical activity at least 3 times per week according to a revised version of the Saltin-Grimby Physical Activity Level Scale (SGPALS) [14].

### Structured examinations and analyses

Body height, body weight, waist circumference, ECG, blood pressure and laboratory analyses including hemoglobin, total triglycerides, total cholesterol, low-density lipoprotein (LDL) cholesterol, high-density lipoprotein (HDL) cholesterol, ApoB/ApoA1 ratio, glycated hemoglobin (HbA1c), blood glucose, potassium, sodium and creatinine were measured as previously described [10].

#### Data from medical records

Information about previous medical history, results of diagnostic examinations, including ECG, echocardiography and coronary angiography, treatments and outcome were collected retrospectively from hospital medical records.

#### Secondary preventive goals

The definition of the secondary preventive goals is based on current guidelines [4] and as follows [10]: blood pressure < 140/90 mmHg, LDL < 1.8 mmol/L, HbA1c < 48 mmol/mol (regardless of diabetes or not), regular physical activity that causes sweating at least twice a week; non-smoking and BMI < 25 kg/m^2^.

#### Statistical analyses

All analyses and data management were performed using SPSS Statistics version 20 (IBM Corp., Chicago, IL, USA). Categorical variables are presented as percentages, and continuous variables are presented as the mean ± SD. For continuous variables, statistical analyses were performed using paired-samples T test and One-way analysis of covariance (ANOVA). Mann–Whitney test was used for non-normally distributed continuous variables. For categorical variables, cross tabulation with Chi-square test was used. All continuous variables were first tested for normality and homogeneity of variance using visual inspection of their histograms and normal QQ plots.

Uni- and multivariable logistic regression analyses were used to study association between the achievement of the secondary preventive goals and the baseline characteristics.

Variables with *p*-value < 0.25 from univariable models were further analyzed in multivariable models. The odd ratios (ORs) from logistic regression analysis were presented with confidence intervals (CIs) (95%) and *p*-values. A *p*-value < 0.05 was regarded as statistically significant.

#### Ethics

The study protocol was approved by the Ethics Committee of the Medical Faculty of the University of Gothenburg and complies with the Declaration of Helsinki. Written informed consent was obtained from each participant. The research assistants signed the Case Report Form, confirming that informed consent was obtained.

## Results

In total 1234 patients hospitalized with AMI between July 2010 and December 2011 were identified; of these, 860 were excluded for such reasons as age > 85 years, not meeting the inclusion criteria or not residing in the Gothenburg area. This information has been described elsewhere [10]. The remaining 374 patients were invited to participate in the study: 56 of these patients declined participation or did not sign the written consent form and 118 did not respond to the invitation. Thus, the final study population included of 200 patients (or 16% of the original sample of 1234 patients) [10].

### Baseline clinical characteristics at hospitalization for index AMI

As shown in in Table 1, baseline data were compared with data from 2 years of follow-up. Of the study cohort (159 were men, 41 women). At baseline the mean systolic blood pressure was 146.8 mmHg (±23.4), the mean diastolic blood pressure 91.3 mmHg (±15.0) and the mean heart rate 75.6 beats/min (±18.8). Prior to the index myocardial infarction, 65.5% of the patients were overweight, 51.0% had hypertension, 26.5% hyperlipidemia and 13.0% had diabetes. A total of 17.0% had a history of ischemic heart disease (IHD) prior to their AMI.

**Table 1:** Baseline characteristics at the time of the index AMI and corresponding values at 2 years of follow-up

	At baseline *n* = 200	At 2 years of follow-up *n* = 200	*P*-values
Age	63.3 ± 9.7	65.5 ± 9,8	
Smoker	45 (22.5)	25 (12.5)	< 0.001
Overweight*	129 (65.5)	144 (72)	< 0.001
Systolic BP (mmHg)	146.8 ± 23.4	137.5 ± 18.0	< 0.001
Diastolic BP (mmHg)	91.3 ± 15.0	79.6 ± 10.3	< 0.001
Heart rate (beats/min)	75.6 ± 18.8	62.1 ± 10.6	< 0.001
Hypertension	102 (51.0)	123 (63.4) (*n* = 194)^#^	0.157
Hyperlipidemia	53 (26.5)	115 (63.5) (*n* = 181)^#^	0.209
Diabetes type1/type2	26 (13.0)	43 (21.8) (*n* = 197)^#^	0.259
Heart failure	6 (3.0)	14 (7)	< 0.001
Atrial fibrillation	8 (4.0)	29 (14.8) (*n* = 196)^#^	0.439
Stroke or TIA	12 (6.0)	14 (7.2) (*n* = 194)^#^	0.319
Renal failure	4 (2.0)	9 (4.5)	< 0.001
Aspirin	199 (99.5)	183 (91.5) (*n* = 199)^#^	0.774
Betablockers	187 (93.5)	166 (84.3) (*n* = 197)^#^	0.452
Statins	193 (96.5)	177 (89.4) (*n* = 198)^#^	0.628
ACE-inhibitors or ARB	177 (88.5)	153 (76.5)	0.463

^*^BMI ≥ 25 kg/m^2^. *BP* Blood pressure, *IHD* Ischemic heart disease, *TIA* Transient ischemic attack, *ACE* Angiotensin converting enzyme, *ARB* Angiotensin receptor blockers. Data are expressed as mean ± SD or *n* (%)

^#^Actual number of patients at 2 years of follow-up

### Follow-up visit at 2 years

The proportion of patients who had been born in Sweden was 79%, 23.5% had a higher education and 36.0% were currently working.

In addition, 87.5% of the whole study cohort did not smoke and 88.5% had a HbA1c < 48 mmol/mol. Only 53% had attained a blood pressure < 140/90 mmHg and 28% had a BMI < 25 kg/m^2^. The secondary prevention goal that was least achieved was optimal lipid control, with only 18.5% of the whole cohort having an LDL < 1.8 mmol (Table 2).

**Table 2:** Achieved secondary prevention goals at the 2-year follow-up after AMI compared with corresponding baseline values

Achieved secondary prevention goals	At baseline *n* = 200	At 2 years of follow-up *n* = 200	*P*-values
Non-smoking, *n* (%)	154 (77.4) (*n* = 199)^#^	175 (87,5)	0.398
Regularly activity*, *n* (%)	No data	91 (45,5)	No data
BP < 140/90 mmHg, *n* (%)	63 (31.5)	106 (53,0)	0.466
HbA1c < 48 mmol/mol, *n* (%)	No data	177 (88,5)	No data
LDL < 1,8 mmol, *n* (%)	9 (5) (*n* = 178)^#^	37 (18,5)	0.096
BMI < 25 kg/m^2^, *n* (%)	67 (33.5)	56 (28)	0.961

^*^Regular physical activity that causes sweating at least two times per week. *BP* Blood pressure, *LDL* Low-density lipoprotein, *BMI* Body mass index

^#^Actual number of patients at baseline

**Fig. 1:**
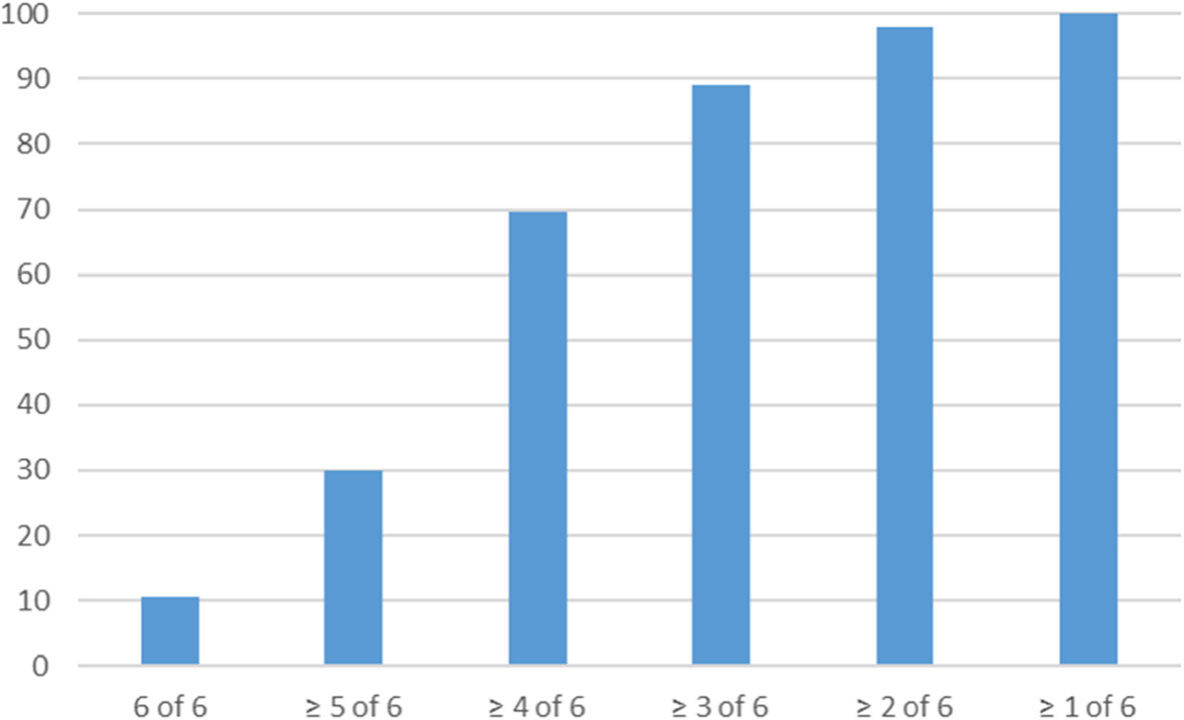
Percentage of achieved goals of guideline recommended secondary prevention 2 years post AMI

Only 21 (10.5%) patients of the total cohort of 200 achieved all six secondary prevention goals (Fig. 1).

### Determinants of suboptimal achievement of secondary preventive goals

Table 3 demonstrates background characteristics of the patient cohort divided into two patient groups according to the number of secondary preventive goals achieved: ≥4 vs. < 4. In the patient group that achieved most goals, 24.5% had a higher education and 37.4% were currently working. The percentage of living alone was 22.3% in the group that achieved the most goals compared to 45.9% in the group that achieved the least goals. A higher proportion of the group with the least achieved goals was born in Sweden compared with the group with the most achieved goals (85.2% vs. 76.3%).

**Table 3:** Patient characteristics at the 2-year follow-up after an acute myocardial infarction: a comparison between patients who achieved > 4 and those who achieved < 4 secondary preventive goals

	< 4 achieved goals *n* = 61	≥4achieved goals *n* = 139	*P*-value
Age, mean ± SD	66.1 ± 9.0	65.2 ± 10.1	0.615
≤ 65 years	31 (50.8)	64 (46.0)	0.541
Demographic variable			
Born in Sweden	52 (85.2)	106 (76.3)	0.120
Living alone	28 (45.9)	31 (22.3)	0.001
Higher education	13 (21.3)	34 (24.5)	0.412
Currently working	20 (32.8)	52 (37.4)	0.632
Clinical status			
Systolic BP (mmHg)	143.3 ± 16.3	135.2 ± 18.1	0.226
Diastolic BP (mmHg)	82.4 ± 11.1	78.7 ± 9.7	0.855
Heart rate (beats/min)	66.7 ± 11.6	60.1 ± 9.5	0.277
Cardiovascular diseases			
Heart failure^a^	4 (6.6)	10 (7.2)	1.000
Atrial fibrillation^a^	4 (6.6)	9 (6.5)	1.000
Stroke or TIA^a^	1 (1.6)	8 (5.8)	0.518
Kidney failure^a^	3 (4.9)	6 (4.3)	0.587

*BP* Blood pressure, *TIA* Transient ischemic attack. Data are expressed as mean ± SD or *n* (%). ^a^Self-specified answers to questions in the SEPAT questionnaire

No common determinants were found in those who failed to achieve six secondary prevention goals according to the European Society of Cardiology guidelines. When analyzing the achievement of each secondary prevention goal separately, each goal had its own predictors (Table 4). Younger [odds ratio (OR) 0.24 (95% CI 0.07–0.74), *p* = 0.013] and unemployed patients [OR 0.23 (95% CI 0.06–0.82), *p* = 0.023] were less likely to be non-smokers. Being born in Sweden [OR 0.28 (95% CI 0.12–0.63), *p* = 0.002] was negatively associated with achieving optimal lipid control.

**Table 4:** Independent predictors of achievement of different secondary preventive goals after AMI assessed by; multivariable logistic regression models

	Odds ratio (95% CI)	*P*-value
Achieved non-smoking		
Sex, male	0.17 (0.02–1.34)	0.093
Age < 65 years	0.24 (0.07–0.74)	0.013
Unemployed	0.23 (0.06–0.82)	0.023
Achieved LDL < 1,8 mmol/L		
Overweight	0.52 (0.24–1.12)	0.096
Native-born	0.28 (0.12–0.63)	0.002
Achieved HbA1c < 48 mmol/mol		
Sex, male	0.46 (0.18–1.18)	0.106
BMI	0.91 (0.82–1.02)	0.095
Achieved regular physical activity		
Sex, male	1.95 (0.93–4.09)	0.078
Living alone	1.94 (1.02–3.69)	0.044
Achieved BMI < 25 kg/m^2^		
Living alone	0.45 (0.20–1.00)	0.050
Diabetes type1/type2	0.21 (0.04–0.98)	0.046
Walking difficulties	0.23 (0.07–0.71)	0.011
Achieved blood pressure < 140/90 mmHg		
Diabetes type1/type2	1.86 (0.61–5.62)	0.271
Heart failure after admission, ≤ 45%	2.77 (1.05–7.31)	0.040
Left ventricular hypertrofi	1.40 (0.57–3.48)	0.466
Age > 65 years	1.49 (0.70–3.20)	0.301
Sex, male	0.36 (0.13–0.97)	0.043

*LDL* Low-density lipoprotein, *BMI* Body mass index. Data are expressed as Odds ratio (95% CI)

Moreover, patients with diabetes [OR 0.21 (95% CI 0.04–0.98), *p* = 0.046] and those with walking difficulties [OR 0.23 (95% CI 0.07–0.71), *p* = 0.011] were less likely to achieve optimal BMI, (Table 4). We also found that living alone [OR 1.94 (95% CI 1.02–3.69), *p* = 0,044] was an independent predictor of achieving regular physical activity.

Finally, when assessing optimal blood pressure control, we found that patients with heart failure diagnosed during hospital admission were more likely to accomplish optimal control [OR 2.77 (95% CI 1.05–7.31), *p* = 0.040]. In contrast, men were less likely to accomplish an optimal blood pressure [OR 0.36 (95% CI 0.13–0.97), *p* = 0.043].

## Discussion

The present study demonstrates that achieving long-term secondary prevention of AMI at 2 years is multifactorial and apparently involves several components that affect this outcome. Accordingly, because of this complexity, we were unable, at least partly, to identify any single determinant common to all six secondary prevention guideline goals.

The importance of smoking cessation in the secondary prevention of cardiovascular heart disease is well established [15], but quitting smoking is a major challenge. In our study many younger patients (≤ 65 years) were smokers. The reason for this can only be speculative. Poor socioeconomic status can be one reason as this has been previously shown to affect the frequency of smoking cessation [16].

Patients with diabetes mellitus were less likely to achieve optimal BMI, perhaps not unexpectedly given that elevated body weight is the predominant cause of type 2 diabetes and the prevalence of obesity and diabetes mellitus among patients with CVD is increasing [12], reflecting an unhealthy lifestyle. Achieving the prevention of CHD includes several lifestyle changes (e.g. a prudent diet and exercising regularly). Accordingly, it is not surprising that patients with mobile aids had difficulty reaching an ideal BMI.

In line with a study showing that heart failure patients with no partner were at higher risk of hospital readmission [17] our study showed a tendency towards significance (*p*-value 0.05), with living alone being an independent predictor of not achieving an optimal BMI. Although these two studies cannot be fully compared, it could be speculated that the support and motivation from a partner may help patients to accomplish recommended lifestyle changes and attain preventive goals. We also demonstrated that living alone was an independent predictor of achieving regular physical activity. Persons living alone may have more healthy, social outdoor activities, prompting them to be more active. Contradictory to the above finding, patients living alone did not reach optimal body weight control despite having increased physical activity. However, successful weight control requires, additional to regular exercise, fewer calorie intake [18].

Our data show that 79% of the overall cohort of patients born in Sweden, were less likely to achieve optimal LDL levels. The reason for this failure to attain optimal LDL levels is unknown and can only be speculative at this time. However, debate in Swedish media about the causal role of LDL cholesterol in coronary disease and the potential harms and ineffectiveness of statins may discourage patients to comply with this recommended medication. Furthermore, in recent years some diets (e.g. low carbohydrates high fat foods) have become popular in Sweden, resulting in a high daily intake of fat and a risk of elevated total cholesterol and LDL. Finally, guidelines for LDL levels have changed significantly over the years, resulting in a lower target level (from 2.5 mmol/L to 1.8 mmol/L).

Men were less likely to achieve optimal blood pressure control. It is well known that the male sex is a risk factor for IHD [19], but this may be more a question of compliance. Our finding is in contrast to other studies showing that men, in comparison with women, have better blood pressure control [20, 21]. However, this may also reflect the fact that optimal blood pressure control is generally very poor [6].

Patients with heart failure at admission with an ejection fraction ≤45% were more likely to achieve better blood pressure control, possibly due to the fact that the failing heart is often associated with lower blood pressure and heart failure medications are mainly neurohormonal inhibitors that all reduce blood pressure.

In Sweden, the national registry of secondary prevention after AMI (SEPHIA) reported that only 26% of patients with AMI fulfilled 4 predefined preventive goals after 1 year [22]. In our study only 10.5% achieved all six predefined secondary preventive goals and only 30% five of the preventive goals after 2 years. This shows that secondary prevention does not improve with time and healthcare efforts seem to be insufficient.

### Limitations

This study has several limitations. First, the study includes a relatively small sample size and only patients from the Gothenburg catchment area were included and therefore may not representative of the general population. However, the patients were consecutively included with pre-specified inclusion and exclusion at our two University hospitals which are the only tertiary referral hospitals in Gothenburg. Second, as the nature of retrospective study there were missing in baseline data as they were obtained by medical records during index hospitalization due to AMI. However, the focus of the present work is to study how well the goals of secondary preventions were achieved 2 years after AMI and what potential underlying factors are. Therefore, the second part of this study was performed by a prospective structured interview at time point of 2-year after AMI in conjunction with blood sampling and physical examinations. This means that, despite some data missing at baseline, data from follow up were both complete and validated. Accordingly, data from baseline and follow-up were obtained by 2 different methodology and therefore not fully comparable.

Third, information from patients who died during the 2 years after the AMI event could not be added because our study was based on personal interview. It is plausible that patients who died within 2 years after their AMI might have had more co-morbidities and serious illnesses, which would result in a final study cohort of healthier patients. Nevertheless, the focus of this study is long-term secondary prevention that requires follow-up visits.

Finally, patients who did not speak fluent Swedish were excluded from the study. It might be assumed that patients living in Sweden who do not speak Swedish fluently have higher rates of unemployment and poorer socioeconomic status.

The strength of this study is our intention to study the goal achievement of long-term secondary prevention in a consecutive hospital cohort from real-world clinical practice, and moreover data were obtained by both personal interview and laboratory analysis as well as physical examination at the same occasion. The personal interview enabled us to capture more precise and detailed information than patient-reported questionnaires.

## Conclusion

In conclusion, our results demonstrates that long-term secondary prevention 2 years after an AMI remains suboptimal, because of multifactorial nature. Accordingly it is hard to define a single determinant for achieving all six secondary preventive goals. Therefore, a tailored and individualized comprehensive assessment and management of risk factors is warranted.

## Data Availability

The dataset supporting the conclusions of this article are included within the article. The datasets during and/or analyzed during the current study available from the corresponding author on reasonable request.

## Abbreviations

ACE: Angiotensin-converting enzyme
AMI: Acute myocardial infarction
ARB: Angiotensin II receptor blockers
BMI: Body mass index
BP: Blood pressure
CHD: Coronary heart disease
CVD: Cardiovascular disease
HDL: High-density lipoprotein
IHD: Ischemic heart disease
LDL: Low-density lipoprotein
TIA: Transient ischemic attack
